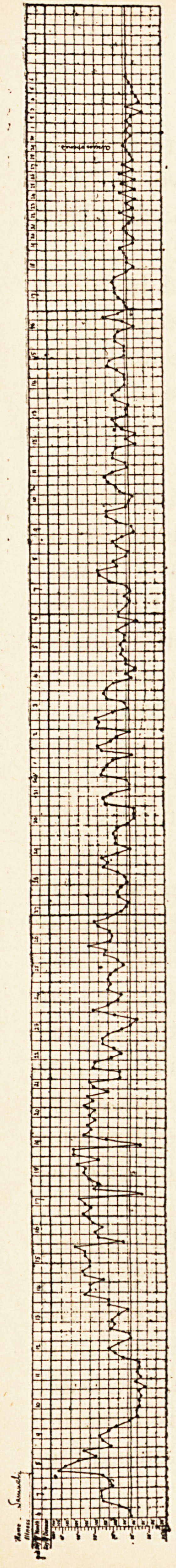# Four Cases of Blood-Poisoning in Brothers

**Published:** 1895-06

**Authors:** F. H. Edgeworth

**Affiliations:** Assistant-Physician to the Bristol Royal Infirmary; Demonstrator of Anatomy at University College, Bristol


### FOUR CASES OF BLOOD-POISONING IN BROTHERS.

BY

F. H. Edgeworth, B.A., M.B. Cantab., B.Sc. Lond.,

*Assistant-Physician to the Bristol Royal Infirmary; Demonstrator of Anatomy at University College, Bristol.*

In the month of August, 1893, four brothers were admitted into the Bristol Royal Infirmary suffering from more or less continued fevers of obscure nature, though probably of common origin. The clinical histories of the patients will first be given, and subsequently the etiology of the observed conditions discussed.

Edward H., aged nine years, was admitted on August 9th, and went home well on October 10th. His mother said he was in good health until five days before, when he complained of pain in the forehead and abdomen, and became feverish and drowsy. Two days after the onset of these symptoms there was some slight epistaxis. He remained in much the same state until admission, on the sixth day of the disease. On examination it was found that the patient was a well-nourished boy, very drowsy and dull, though answering rationally any questions that were put to him. He complained of frontal headache and of pain in the umbilical region. The temperature was 101·4°. The skin was of a sallow hue, but there was no jaundice. The tongue was moist, with a slight white fur. The heart and lungs were normal. There was no abdominal distension or tenderness, or enlargement of the liver or spleen. The urine was passed in fair quantity, of a pale yellow colour and acid reaction, and contained no albumen. No change of colour occurred on boiling the urine or on adding nitric acid to the cold urine; but on boiling it with a little nitric acid, a marked purplish-red colour developed. There was no diazo-reaction, or evidence of bile-pigments. The bowels had not been opened for several days; on administration of an enema, it was found that the fæces were "formed" and of a brownish colour.

The illness was acute, as shown in the chart; the temperature remained high, with slight fluctuations, until the early part of September, when it was about 103° for over a week, and then gradually subsided. There was never any abdominal distension or typhoid spots, or enlargement of the spleen or liver, or light-yellow stools. The bowels were only opened by enemata every two days or so, and the stools were always semi-solid and brownish. The boy lay in a drowsy lethargic condition, sleeping a good deal, not delirious even at night, taking his food (milk and beef-tea) moderately, and gradually getting thinner until about the middle of September, when as the fever lessened he slowly became better and at last quite well. By physical examination no lesion could be detected in any of his organs, save an extreme heart-weakness and the peculiar characters of the urine mentioned above.

Fred. H., aged six years, was admitted on August 6th, and went out well on September 12th. He was under the care of Dr. Watson Williams, who has kindly given me permission to refer to his case. He was said to have been taken ill somewhat suddenly a fortnight before admission, with headache and drowsiness. On examination it was found that the patient was drowsy and dull, with a sallow skin, but no jaundice. The tongue was moist and coated. The thoracic organs were normal. The abdomen was not distended or tender, nor was there any enlargement of the spleen or liver. The urine presented the same features, though less marked, as in the case just related. The bowels were confined; on being opened by an enema, the fæces were solid and brownish. The temperature was 99°. The temperature chart shows that there was practically no fever. The boy steadily improved, and finally went out quite well. No physical signs of any local lesion were detected throughout the illness.

These two are uncomplicated cases: the first, severe; the second, slight. In the other two brothers additional features appeared.

William H., aged seventeen, was admitted on August 6th; went out well on September 19th. Early in July the patient began to experience a feeling of nausea, which, though constant, was generally worse after exertion. This was soon accompanied by great languor and debility, which gradually increased, so that on July 16th the patient was so weak that he had to take to bed, and remained there in much the same condition until admission on August 6th. On examination it was found that the patient was 5 ft. 10 in. in height, 9 stone 3 lbs. in weight, and somewhat emaciated. He complained chiefly of extreme weakness, and was dull and drowsy, although not nearly to the same extent as his brothers. The skin was of a sallow hue, and there was no jaundice. Numerous small purpuric spots were scattered over the whole body, and there was a large one on the left side of the abdomen. The lips were parched and covered with dry mucus, and the tongue was thickly coated with a white fur. The heart-sounds were normal, though very feeble; and the cardiac impulse, which was normal in position, and the radial pulse, were very weak—88 per minute. The lungs were normal. There was no distension or tenderness of the abdomen, the liver was normal in size, the spleen was slightly enlarged. The urine was passed in fair quantity, of specific gravity 1005, alkaline in reaction, of a bright-red colour; it contained a great many red blood-corpuscles, with a few blood-casts, white corpuscles, and triple phosphate crystals. The temperature was 102°.

The course of the temperature during the illness is shown on the chart. It was found, on measuring the urine, that a large quantity was passed—from sixty to ninety ounces daily. The red blood-cells and blood-casts persisted and did not grow less until the latter end of August, when they gradually diminished and finally disappeared. On August 19th the marked sallow colour of the skin had almost faded, and the purpuric spots had disappeared. The bowels were still confined; they were opened by enemata, the motions were solid and of a brownish colour. The enlargement of the spleen had also subsided. The patient however was still very weak, and the cardiac impulse and pulse were very feeble. The patient very slowly improved: after a time he was able to sit up a little and eat more food; he gradually grew stronger, and at last well.

The last case was that of Samuel H., aged thirteen years, who was admitted on August 6th, and was transferred to the care of Mr. Prichard on September 28th. His illness was said to have begun about three weeks before admission, with heaviness and headache and slight diarrhœa. On admission he was found to be emaciated, anæmic, and very deaf, continually moaning, though apparently not in any pain. There was a discharge of pus from the left ear, which was said to have been present since an attack of measles some years before. The cardiac sounds and impulse were very weak; the pulse was ninety-six per minute, feeble, of low tension and slightly irregular. The lungs were normal. There was no distension or tenderness of the abdomen. The liver was normal in size and the spleen slightly enlarged. The tongue was moist and slightly coated, the bowels were confined. The urine was acid in reaction, of specific gravity 1020, light-yellow in colour, and contained no albumen; on boiling it with a little nitric acid, a purplish-red colour appeared, exactly as in the first two cases related. The temperature on admission was 98·8°. The course of the fever is shown in the chart. The boy remained in much the same condition during the whole month of August; the only fresh feature being that on the 17th a pustular rash appeared on the shoulders, neck, and backs of arms and hands: this disappeared in about ten days, a few of the larger pustules having to be opened by incision. Throughout the illness the bowels were confined and were opened only by enemata, the fæces were solid and brownish. About the middle of September the patient appeared to be in pain in the left buttock, and a little later on distinct fluctuation was detected. Mr. Prichard saw the boy and diagnosed an abscess, and on the 28th the patient was transferred to his care. The abscess was opened, and proved to be periarticular, round the hip-joint, which was normal. The abscess rapidly healed, and the patient went home well on October 9th.

The above is a short account of the main clinical features of these cases. The family history was good; the father and mother were well, as were also two sisters and one other brother. The oldest patient worked with his father, a carpenter; the second went out to work elsewhere, whilst the two younger patients went to school.

Dr. Davies kindly saw the patients with me and had their home examined. He reported that the house the patients lived in was in a good sanitary condition, as were also the workshops where the two older patients were employed, and the school the two younger ones attended; also that there was no typhoid fever in the neighbourhood.

On comparing the cases, it is obvious that William H. (the oldest, who had hæmaturia and purpura) and Samuel H. (who had a pustular rash and a periarticular hip-abscess) were gradually taken ill about the end of the first week of the almost tropical month of July, 1893, with a continued fever, gradually increasing bodily and cardiac weakness, a gradually oncoming mental apathy and languor, and an increasing sallowness of skin; whilst the two younger patients became rather more suddenly ill about the end of the month, with much the same symptoms. These symptoms existed in all four brothers at the time of their admission in a marked degree (with the exception that in the case of Fred. H., the youngest, the fever was very slightly marked) and persisted throughout, only gradually disappearing as they slowly recovered. Again, in all four brothers there was a considerable amount of blood-destruction, evidenced by the pallor and by the diminution of the number and quality of the red corpuscles. Thus, in Samuel H., on August 28th, the red corpuscles were 58 per cent., and the hæmoglobin 40 per cent., of the normal. And the same features, in a less marked degree, existed in the blood of the other brothers.

If the two uncomplicated cases only (Edward and Fred.) had been admitted, it would have been very difficult, if not impossible, to make any diagnosis; but the complications in the other two throw some light on the question. In the case of William, there was on admission a purpuric rash and hæmaturia, the latter of which persisted for nearly a month. The blood in the urine was not due to a nephritis, as shown by the large quantity of urine, but certainly came from the upper part of the urinary tract, since it was intimately mixed with the urine, and some of the blood at any rate had passed through the renal tubules, for blood-casts were present, though few in number. Again, in the case of Samuel there was a well-marked rigor, with rise of temperature to 106° on the day after admission, ten days later a pustular skin rash, and subsequently a periarticular hip abscess.

These phenomena suggested that the cases were some form of blood-poisoning. I therefore asked Mr. Stoddart to examine the blood for micro-organisms, which he was good enough to do. He reported that in none of the four cases were any to be found. Streptococci and staphylococci were of course discovered in the pus from the pustular rash of Samuel.

In the case of the three brothers in whom there were no secondary abscesses, and no evidence of micro-organisms in the blood, one is not strictly entitled to say more than that they were cases of sapræmia, of poisoning by products of micro-organisms, whilst the case of Samuel may rigidly be termed one of pyæmia. This distinction, however, appears much too arbitrary when the numerous points of similarity in the cases are considered, and it is much more probable that they were all cases of infection, although in the few drops of blood examined no organisms were found.

In reference to this, it is worth noticing that it was precisely in these two brothers, Edward and Samuel, who had presumably been longest exposed to the source of infection—lying at home ill for four and three weeks respectively before being admitted,—that complications occurred ; viz., hæmaturia and purpura in the one, abscesses in the other, and slight enlargement of the spleen in both. Whilst the other two brothers—admitted within a fortnight of being taken ill—were simple cases.

It is somewhat remarkable that no primary lesion was found in any one of the four cases: this is, however, paralleled by many instances of pyæmia ; *e.g.*, in malignant endocarditis there is, as a rule, no lesion discoverable which might indicate the place of bacterial invasion. The existence of a peculiar pigment—or rather aromatic substance—in the urine of the three younger brothers possibly affords a clue. The urine in each case was of a light-yellow colour, and on boiling with a little nitric acid became reddish-violet. This was due to a skatoxyl-chromogen.[1] Now, skatol is formed by putrefaction of proteids in the intestine. Perhaps then the bacterial invasion was by way of the intestinal tract.

It might be suggested—indeed, it was suggested by some who saw them—that the patients were suffering from typhoid fever. One could not deny the possibility of this in the case of those two brothers (William and Samuel) who were admitted three weeks or more after being taken ill; though against the theory it may be said that there was no positive physical sign of that disease. The courses of the temperatures are not consistent with a relapse or relapses, which must be assumed; and whilst abscesses, especially periosteal ones, may occur as sequelæ, yet purpura and hæmaturia are unknown in typhoid fever. Further, the other two brothers (Edward and Fred) were admitted within a fortnight of falling ill, and in them there was no enlargement of the spleen, no distension of the abdomen, no typhoid spots, no typhoid stools, no bronchitis, and no typhoid facial expression. Also there was no diazo-reaction in the urine —which is of some importance; for though the diazo-reaction has no positive value, being found, *e.g.*, in measles and general tuberculosis, yet its absence in the second week of a supposed typhoid fever is a very great, if not absolute, indication that the illness is not that disease. If, then, it be concluded that these two brothers did not suffer from typhoid fever, it may be considered certain that the other two, who presented the same symptoms, together with complications, also did not.

One is apt, no doubt, in any case of continued fever lasting for a fortnight without any symptoms and physical signs other than those which might be due to the heightened temperature, to assume that it is one of typhoid fever or general tuberculosis. These cases are good instances showing that such a diagnosis may occasionally be incorrect.

[1] See Halliburton, *Chemical Physiology*, 1891, pp. 744–5.

## Figures and Tables

**Figure f1:**
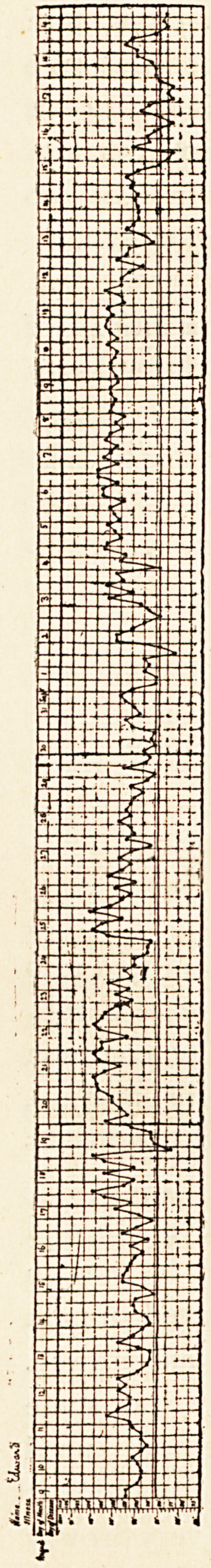


**Figure f2:**
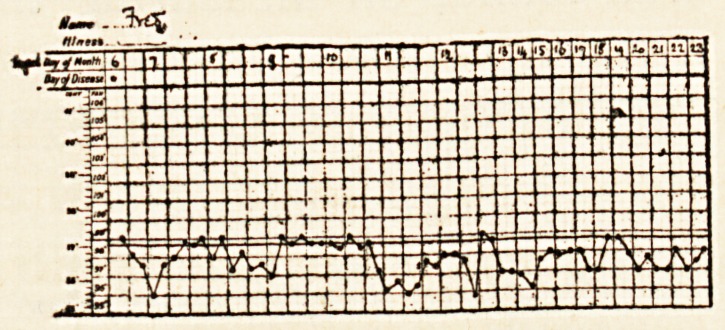


**Figure f3:**
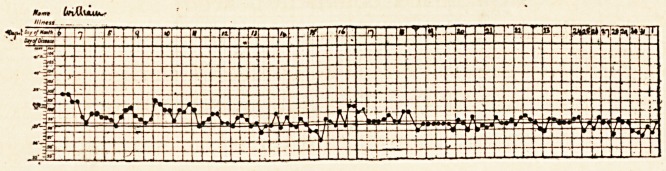


**Figure f4:**